# Supplementary material for: Protocol to Assess the Knowledge, Attitude, and Practices of Midwives in the Implementation of Maternal Healthcare Guidelines in a Selected District, Limpopo Province, South Africa
Source: Nurs Rep. 2025 Oct 15;15(10):368. doi: 10.3390/nursrep15100368 (PMC12566797; doi:10.3390/nursrep15100368)
Supplement: Supplementary file 1 [file nursrep-15-00368-s001.zip › nursrep-3731395-supplementary.pdf]

Supplementary Materials: questionnaire for data collection.

SECTION A (BIOGRAPHICAL DATA)

1. How old are you?

20-30 years	31-40 years	41-50 years	51-60 years	60+ years

2. What is your race group?

African	White	Indian	Coloured	Other

3. What is your gender?

Male		Female		Other	

4. What is your nationality?

South African	Other. Please specify.

5. How long have you been working as a midwife?

0–5 years	5–10 years	10–15 years	15–20 years	>20 years

6. Have you ever been exposed to the South African maternal health guideline?

Yes		No	

SECTION B

This section is about the knowledge of midwives in the implementation of maternal healthcare guidelines during their practice in their primary care facilities.

1. Do you implement maternal healthcare guidelines during your practice?

Yes		No	

2. Have you received formal training on maternal healthcare guidelines?

Yes		No	

If yes, on which year? ______________________

3. Do you have the latest maternal healthcare guidelines in this facility?

Yes		No	

4. How often do you consult maternal healthcare guidelines in your practice?

Always	Often	Sometimes	Rarely	Never

5. In what year did the latest maternal health guidelines were released?

2016	2018	2020	2022	2024

6. Failure to implement maternal healthcare guidelines leads to an increase in the maternal mortality rate.

Strongly agree	Agree	Neutral	Disagree	Strongly disagree

7. Failure to implement maternal healthcare guidelines does not lead to an increase in maternal mortality rate.

Strongly agree	Agree	Neutral	Disagree	Strongly disagree

8. I encounter problems when reading maternal healthcare guidelines.

Strongly agree	Agree	Neutral	Disagree	Strongly disagree

9. If you encounter problems when reading the maternal healthcare guidelines, please describe those problems.

10. Maternal healthcare guidelines are easy to use.

Strongly agree	Agree	Neutral	Disagree	Strongly disagree

11. Maternal healthcare guidelines are easy to use even during emergencies.

Strongly agree	Agree	Neutral	Disagree	Strongly disagree

SECTION C

This section is about the attitudes and practices of midwives in the implementation of maternal healthcare guidelines during their practice in their primary healthcare facilities.

1. I can perform my daily duties well without making use of maternal healthcare guidelines.

Strongly agree	Agree	Neutral	Disagree	Strongly disagree

2. The maternal healthcare guidelines books are not enough for all midwives.

Strongly agree	Agree	Neutral	Disagree	Strongly disagree

3. The shortage of personnel contributes to poor implementation of maternal health guidelines.

Strongly agree	Agree	Neutral	Disagree	Strongly disagree

4. The lack of infrastructure contributes to poor implementation of maternal health guidelines.

Strongly agree	Agree	Neutral	Disagree	Strongly disagree

5. I need in-service training on how to properly implement maternal healthcare guidelines.

Strongly agree	Agree	Neutral	Disagree	Strongly disagree

6. Maternal healthcare guidelines are time-consuming and not easy to use during emergencies.

Strongly agree	Agree	Neutral	Disagree	Strongly disagree
